# Supplementary material for: Enhancing Vietnamese Students’ Acceptance of School Lunches Through Food Combination: A Cross-Over Study
Source: Nutrients. 2025 Apr 20;17(8):1385. doi: 10.3390/nu17081385 (PMC12030064; doi:10.3390/nu17081385)
Supplement: Supplementary file 1 [file nutrients-17-01385-s001.zip › nutrients-3569569-supplementary.pdf]

Supplementary Material

Table S1. Five new menus developed from five current menus

	Current menu			New menu		
	Dish	Ingredient		Dish	Ingredient	
Monday	**Rice**	Rice	90g	**Rice**	Rice	90g
	**Fried chicken**	Chicken wings	100g	**Fried chicken**	Chicken wings	100g
				**Stir-fried vegetables**	Cabbage	40g
					Carrot	15g
					Leaf mustard	10g
	**Soup**	Tomato	58g	**Soup**	Tomato	23g
		Bean sprouts	40g		Bean sprouts	10g
		Tofu	15g		Tofu	15g
		Pork	3g		Pork	3g
		Spring onion	2g		Spring onion	2g
Tuesday	**Rice**	Rice	90g	**Mixed rice**	Rice	90g
					Carrot	28g
					Chayote	11g
					Leaf mustard	8g
	**Braised pork with tofu**	Minced pork	68g	**Braised pork with tofu and radish**	Minced pork	68g
		Tofu	44g		Tofu	44g
					Radish	20g
	**Soup**	Sauropus leaf	100g	**Soup**	Sauropus leaf	24g
		Minced pork	6g		Minced pork	6g
					Sponge gourd	9g
Wednesday	**Rice**	Rice	90g	**Rice**	Rice	90g
	**Fish ball**	Fish ball	100g	**Fish ball**	Fish ball	100g
		Ketchup	10g		Tomato	6g
		Spring onion	2g		Onion	2g
					Carrot	4g
					Spring onion	2g
				**Stir-fried vegetables**	French beans	18g
					Carrot	10g
					Chayote	18g
	**Soup**	Spinach	98g	**Soup**	Spinach	40g
		Minced pork	3g		Minced pork	3g
Thursday	**Rice**	Rice	90g	**Rice**	Rice	90g
	**Braised shrimp and pork**	Minced pork	75g	**Braised shrimp and pork**	Minced pork	75g
		Shrimp	31g		Shrimp	31g
		Spring onion	2g		Spring onion	2g
				**Boiled-salad**	Cabbage	24g
					Carrot	10g
					Broccoli	17g
					Corn	11g
					Sesame dressing	10g
	**Soup**	Pumpkin	98g	**Soup**	Pumpkin	36g
		Minced pork	3g		Minced pork	3g

	Current menu			New menu		
	**Dish**	**Ingredient**		**Dish**	**Ingredient**	
**Friday**	**Yangzhou Fried Rice**	Rice	80g	**Yangzhou Fried Rice**	Rice	80g
		Carrot	8g		Carrot	8g
		French bean	9g		French bean	9g
		Egg	6g		Egg	6g
		Chinese sausage	10g		Chinese sausage	10g
		Sausage	10g		Sausage	10g
		Minced pork	10g		Minced pork	10g
		Spring onion	2g		Spring onion	2g
				**Stir-fried vegetable**	Cabbage	25g
					Tomato	15g
					Cucumber	6g
					Sesame dressing	5g
	**Soup**	Water spinach	52g	**Soup**	Water spinach	20g
		Bean sprouts	29g		Bean sprouts	15g
		Minced pork	3g		Minced pork	3g
